# Integration Models for Delivering COVID-19 Vaccines Through HIV Services in Low-and Middle-Income Countries: A Scoping Review

**DOI:** 10.3390/idr17060146

**Published:** 2025-12-05

**Authors:** Nyanyiwe Masingi Mbeye, Roselyn Chipojola, Susan Banda, Prince Kaude, Aaron Mdolo, Charles Nwosisi, Sandra Mounier-Jack

**Affiliations:** 1 Evidence Informed Decision-Making Centre, School of Global and Public Health, Kamuzu University of Health Sciences, Blantyre P/Bag 360, Malawi; rchipojola@kuhes.ac.mw (R.C.); sbanda@kuhes.ac.mw (S.B.); pkaude@kuhes.ac.mw (P.K.); 2 United Nations Children’s Fund (UNICEF) Malawi, Lilongwe P.O. Box 30375, Malawi; amdolo@unicef.org (A.M.); cnwosisi@unicef.org (C.N.); 3 United Nations Children’s Fund (UNICEF) Headquarters, 3 United Nations Plaza New York, New York, NY 10017, USA; sandra.mounier-jack@lshtm.ac.uk

**Keywords:** corona virus infections, human immunodeficiency virus, vaccination, health service delivery, developing countries

## Abstract

Background: The Coronavirus Disease 2019 (COVID-19) remains a major global public health issue. People living with HIV (PLHIV) are among the vulnerable groups facing a higher risk of severe outcomes. Combining COVID-19 vaccination with HIV services can improve access and utilization of the vaccine among PLHIV although effective methods of delivery are yet to be ascertained. We conducted a scoping review to identify and describe models for delivering COVID-19 vaccines through HIV care services in low- and middle-income countries (LMICs). Methods: We used PRISMA-ScR guidelines to conduct the review. On 3rd and 4th February 2025, we searched PubMed, Web of Science, Cochrane Library, and EMBASE for studies on integrated COVID-19 vaccine delivery for PLHIV. Results: Three studies from sub-Saharan Africa reported call-back strategy, diverse partnership, and mixed service delivery models for implementing COVID-19 vaccination in HIV care services. Key strategies that were used included building capacity, generating demand, managing the supply chain, and involving stakeholders. The outcomes showed significant increases in vaccination coverage among PLHIV and reduced vaccine wastage. Conclusions: Integrating COVID-19 vaccination into HIV services is practical and effective in LMICs. It makes use of current infrastructure, partnerships, and local innovations.

## 1. Introduction

The Coronavirus Disease 2019 (COVID-19) is still a public health threat, even though the World Health Organization (WHO) no longer classifies it as a public health emergency of international concern [1]. The pandemic deeply affected nearly every part of society, including governments, healthcare systems, and global economies [2]. While various non-drug methods helped slow the spread of the virus, vaccination remains the main strategy for controlling COVID-19 [3]. Many studies have shown that traditional mRNA vaccines effectively prevent severe symptomatic infection, hospitalization, and death and are safe and effective across different populations [4,5].

At the start of the vaccine rollout, the WHO’s COVID-19 vaccine prioritization roadmap suggested focusing on reducing illness and death, especially with limited vaccine supply starting with healthcare workers and other frontline personnel due to their increased exposure and essential roles in responding to the pandemic [6]. Despite being an understandable approach, it unintentionally ignored the broader view of health as a basic human right, especially for other vulnerable groups. Evidence consistently shows that people aged 65 and older and those with existing medical conditions (comorbidities) are at much greater risk of getting COVID-19, experiencing long hospital stays, and facing worse health outcomes [7,8,9,10]. Several studies have also shown that people living with HIV are at higher risk of severe COVID-19 infections, qualifying them a priority group for preventive measures including vaccination [11,12]. While many PLHIV are willing to get the COVID-19 vaccine, the actual uptake has not been satisfactory [13]. Several reports show strong evidence that COVID-19 vaccines are safe for people living with HIV (PLHIV), with mostly mild, self-limited adverse events and good immunogenicity, particularly after booster doses [14,15,16,17]. Despite this, there is still a significant gap in specialized vaccine delivery strategies for PLHIV. Most COVID-19 vaccination models, like mass vaccination sites, mobile clinics, fixed-post immunizations, and home services, were designed with the general population in mind [18,19] leaving the specific needs and access challenges faced by PLHIV unaddressed.

Recently, some countries have started incorporating COVID-19 vaccine delivery into regular healthcare services [20]. For instance, Heidari et al. describe models where vaccination clinics are located alongside services such as needle exchange programs, HIV and Sexually Transmitted Infections (STI) testing for people who inject drugs, and antenatal care visits for pregnant women at 20 weeks [21]. These integrated methods have improved vaccine access, especially by reducing the logistical and financial burdens related to transportation, childcare, and time off work. In addition, regular contact with healthcare providers and vaccine counseling in these settings have helped build trust and increase vaccine confidence [20,22]. Moreover, health systems facing critical staffing gaps often find it hard to offer separate COVID-19 vaccination services as vaccine supply improves and efforts to reach people unfamiliar with adult vaccination programs have generally faced setbacks due to vaccine hesitancy, misinformation, and ongoing structural obstacles [23].

To effectively tackle vaccine distribution for people living with HIV and enhance health outcomes, identifying and mapping the existing models used to deliver vaccines to this population is essential. Understanding these methods can help policymakers and healthcare providers design better research and implementation strategies. Furthermore, gaining a deeper insight into these models may offer helpful guidance on how future vaccine delivery and its integration into primary healthcare and vaccination programs can be improved, particularly in LMICs. This scoping review explored existing COVID-19 vaccine integration models in LMICs focusing on approaches relevant to PLHIV.

## 2. Materials and Methods

### 2.1. Study Design

We carried out a scoping review of the literature according to the Preferred Reporting Items for Systematic Reviews and Meta-Analyses extension for Scoping Reviews (PRISMA-ScR) guidelines [24]. We performed a systematic search of the literature from 03 to 04 February 2025 across four electronic databases: PubMed, Web of Science, Cochrane Library, and EMBASE. The scoping review protocol was registered in the International Platform of Registered Systematic Review and Meta-analysis Protocols (INPLASY), registration number: INPLASY2025100010; DOI: 10.37766/inplasy2025.10.0010 [25].

### 2.2. Eligibility Criteria

Inclusion and exclusion criteria were defined using a modified Population, Intervention, Comparison, Outcomes (PICO) framework focusing on studies involving PLHIV, men and women aged 18 and older residing in LMICs. Since our main interest was on how COVID-19 vaccination is integrated into HIV care services, we included studies that reported on strategies or models for delivering COVID-19 vaccines through HIV care platforms. Eligible study designs included all empirical research approaches: quantitative, qualitative, and mixed methods such as observational studies, randomized controlled trials, quasi-experimental studies, cohort and case-control studies, and single-case reports. We excluded studies if they were conducted outside of LMICs or did not focus on vaccine integration specifically for PLHIV.

### 2.3. Information Sources

We developed a thorough search strategy using Medical Subject Headings (MeSH) and Boolean operators. This strategy combined terms related to PLHIV, COVID-19 vaccines, and integration strategies. The search terms and strategies were based on an initial scoping search and consultations with experts. Full details of the search strategy and the databases used are provided in Appendix A.

### 2.4. Selection Process

Two reviewers, PK and SB independently carried out the screening and review process using Rayyan, an online tool for managing systematic reviews. Any differences in opinion were resolved through discussion or with input from third reviewers, NMM or RC. In the first screening phase, we assessed titles and abstracts for eligibility. We then performed full-text screening on all records that met the inclusion criteria. We documented the reasons for excluding studies during the full-text review stage.

### 2.5. Data Extraction and Synthesis

We extracted data from the included studies using a standardized form created by the authors and summarized it in a table. The structured extraction included study characteristics, model type, model features, strategies, outcomes, and lessons learned. We performed a narrative synthesis with tabulation to analyze the data.

## 3. Results

### 3.1. Study Selection

**Figure 1:**
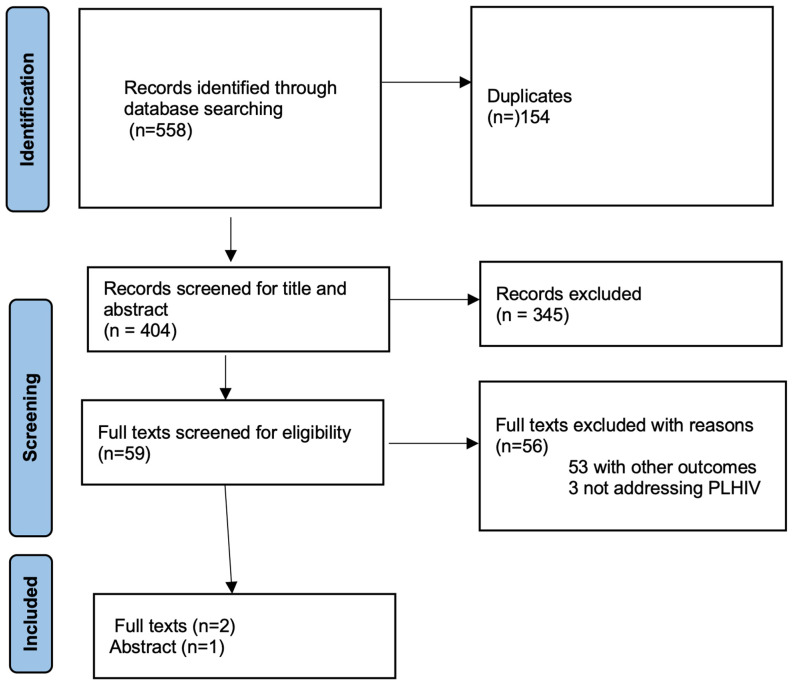
PRISMA Flow Diagram.

The search resulted in 558 records. After removing 154 duplicates, 404 records were left for title and abstract screening. From these, 59 records were found to be potentially relevant and were reviewed in full text. Ultimately, three studies met the inclusion criteria and were part of the final analysis as shown in Figure 1 below.

### 3.2. Characteristics of the Included Studies

All included studies were from sub-Saharan Africa, specifically Tanzania (*n* = 2) and Zambia (*n* = 1) as shown in Table 1. They focused on people living with HIV, their families, and healthcare workers. The Tanzanian studies [26,27] included a case study and an abstract presented at the National Health Summit. The Zambian study was a report from the national COVID-19 vaccination campaign [23].

### 3.3. Integration Models Used for COVID-19 Vaccination and HIV Services in Low- and Middle-Income Countries

The reviewed studies identified three distinct models for integrating COVID-19 vaccine delivery with HIV care services. These include the call-back strategy, the diverse partnership model, and a mixed service delivery model as explained in Table 2 below.

## 4. Discussion

To our knowledge, this is the first review to focus on models and/or strategies for integrating COVID-19 vaccination with HIV care and services in low- and middle-income countries. Two included studies successfully applied common themes that contributed to their effectiveness [23,26].

The mixed service delivery model used in Zambia focused on planning and coordination. It aimed to use existing in-country systems, programs, and resources for COVID-19 vaccination through joint planning with the Ministry of Health, funding organizations, and provincial representatives. Similarly, the diverse partnership model in Tanzania highlighted cooperation and coordination as key factors for improving vaccine uptake. Strong collaboration between healthcare providers and public health agencies is recognized as crucial for successful health programs and interventions [29,30].

Building capacity was a major theme in all the studies. This included promoting vaccine awareness and education to combat misconceptions, as well as offering incentives to meet vaccination goals. Community health workers (CHWs) served as important links between healthcare workers and communities, while training healthcare workers in community mobilization and service delivery helped address the challenges of static service models. This method increased access by providing vaccines in public locations like markets, malls, and churches. Past studies have also emphasized the important role of community mobilization in improving intervention acceptance, enabling communities to take charge, and encouraging social and behavior change through peer support and local solutions [31,32,33,34,35].

Effective integration required solid logistics and supply chain systems along with clear monitoring and evaluation methods. Both models focused on making sure there was a steady vaccine supply for district-level populations, using existing infrastructure for vaccine storage when possible. Research suggests that single-dose vaccines could increase coverage, and that strong involvement from stakeholders is necessary to support vaccine purchase, transportation, and distribution [36]. Also, reliable supply chains for health commodities have shown to be essential to strong health systems and are important for reaching national and regional health security goals [37].

The call-back strategy was consistently used across all models to increase vaccine acceptance and uptake among people living with HIV. This strategy involved creating targeted promotional materials that highlighted the increased risk of severe COVID-19 illness for this group. Regular patient reminder calls informed individuals about vaccine availability at HIV clinics, while use of patient champions helped reach PLHIV within their communities. Evidence supports the effectiveness of the call-back approach in enhancing patient understanding, maintaining treatment plans, and reducing unnecessary healthcare visits [38].

### Limitations

This scoping review focuses on a topic that is very important for public health. However, the studies included are the lowest level in the hierarchy of evidence and are only relevant to the African context. The lack of higher-level evidence, like systematic reviews or randomized controlled trials, limits how far we can apply our findings beyond the specific areas we looked at. Even with a thorough search strategy, we found no eligible studies from outside Africa, which restricts the geographic range of the review. Deliberate strategies such as collaborating with international HIV and immunization programs could help access data and insights from underrepresented regions. This could help expand research coverage despite acknowledged limitations. Additionally, future research should aim to fill evidence gaps in regions outside sub-Saharan Africa.

## 5. Conclusions

This scoping review shows that incorporating COVID-19 vaccination into trusted health platforms, especially HIV services, can significantly increase uptake among high-risk groups like people living with HIV in low- and middle-income countries. The reviewed models were successful by including vaccination in familiar care pathways, empowering healthcare workers to adjust delivery at the facility level and encouraging equitable access through community involvement and focused outreach. These methods strengthened the connection between vaccine demand and supply while reducing waste and leveraging the trust patients have in their providers.

The experiences from Tanzania and Zambia highlight that innovation tailored to specific contexts, along with strong partnerships across political, government, religious, and community areas, can achieve rapid and lasting improvements, even in resource-limited environments. Expanding these models will need ongoing investment, political support, and a willingness to adjust to future public health challenges.

### Recommendations for Future Vaccine Implementation

Based on the lessons learnt in this review, we suggest the following priority actions for policymakers, implementers, and partners in other LMICs to adapt or test:

1. Using existing health platforms such as building on established systems like HIV clinics and community health programs is critical for effective and sustainable vaccination integration.
2. Provide ongoing training for healthcare workers in vaccine delivery, community engagement in order to address vaccine hesitancy.
3. Improve Community Engagement and Education to ensure use of culturally relevant communication strategies with community leaders, patient advocates, and civil society in order to boost vaccine awareness and uptake.
4. LMICs should aim to implement reminder and follow-up systems such as using call-back and other reminder methods to reduce missed opportunities and increase completion rates, particularly among PLHIV.
5. Remove barriers to ensure equitable access. This could be achieved by providing transport reimbursements, mobile services, and flexible hours to address logistical and economic challenges. Effective partnerships with funders and other organizations would be key in supporting these initiatives.
6. Strengthen data systems and monitoring to provide strong monitoring and evaluation systems for tracking coverage and identifying gaps.
7. Consider fostering multisectoral partnerships through engaging with government agencies, Non-governmental Organizations (NGOs), religious and political leaders, and donors to facilitate coordinated resources, advocacy, and policy support.
8. Plan for strong supply chain for keeping a steady vaccine supply that would likely avoid stockouts and waste especially in remote areas.
9. Encourage use of implementation science to assess and expand new methods like mixed service delivery and community-based vaccination.
10. Preparing for future health emergencies such as building rapid integration and adaptation processes within health systems would lead to quick response to emerging infectious disease threats.

## Figures and Tables

**Table 1:** Summary of the included Studies.

Title	Author	Year	Country	Study Type	Setting	Target Population	Age	Integration Model	Strategies Used	Outcomes	Lessons Learnt
Accelerating COVID-19 Vaccination Among People Living with HIV and Health Care Workers (HCWs) in Tanzania: Case Study	Jalloh et al. [26]	2024	Tanzania	Case study	562 high-volume HIV clinics	PLHIV and HCWs	≥20 years	Diverse partnership model	Training HCWs to increase certified vaccinators. Establishing vaccination sites in high-volume HIV clinics with strong regional health authority support. Engaging HCWs to address their concerns and build their vaccination confidence. Strengthening capacity of HCWs and community health workers (CHWs) to facilitate vaccination in facility- and community-based settings. Monitoring uptake of COVID-19 vaccination in high-volume facilities.	**Increased vaccination uptake:** the proportion of fully vaccinated adult PLHIV increased from <1% to 97% and the proportion of fully vaccinated HCWs increased from 23% to 80% in the monitored facilities between September 2021 and September 2022.	Building strong partnerships with political, government, and religious leaders to support planning, capacity-building, demand creation, and service delivery that would enhance vaccine reach and acceptance across all levels. Use of innovative strategies to address gaps in knowledge, supply, and service delivery such as incentives, peer champions, integration with other health services, community outreach at mass gatherings, and live media coverage. Proper planning to forecast demand, manage data entry, and minimize vaccine wastage.
Leveraging HIV Program and Civil Society to Accelerate COVID-19 Vaccine Uptake, Zambia	Bobo et al. [23]	2022	Zambia	National Vaccination campaign	HIV treatment centres	PLHIV and their family members	Not reported	A mixed service delivery model	Engaged HIV treatment centers by training existing staff to provide vaccinations and promoting staff vaccination uptake. Developed targeted materials for people with HIV at high risk of severe illness and scaled models nationally. Engaged civil society leaders to endorse vaccination and expanded service delivery by integrating community-based models from successful HIV programs. Adapted strategies from Zambia’s successful childhood immunization program.	**Increased COVID 19 vaccination coverage in Zambia** (Reported outcomes for all COVID-19 vaccinations in Zambia not only PLHIV)	**Planning and coordination:** Leveraging existing in-country systems/programs/resources for COVID-19 vaccination. Engaging national, provincial, and district health bodies from the outset. Developing district-level micro plans based on standard tools that are approved at provincial level and achieved by lower levels (i.e., district health offices, service delivery teams). Using joint planning by Ministry of Health, funding organizations, and provincial representatives. Establishing centralized Monitoring and Evaluation (M&E) tools for national tracking of progress. Beginning with a small pilot in a few sites and rapidly iterate to improve quality, using a continuous quality-improvement approach. Rapidly scaling-up successful practices to quickly enhance effect. Developing targets that can be implemented and achieved by lower levels (i.e., district health offices, service delivery teams). **Service delivery:** 9. Adequately capacitating HCWs in HIV, Maternal and Child Health (MCH), and other clinics to deliver COVID-19 vaccines. 10. Investing in community mobilization and service delivery to overcome limits of a static service delivery approach and reach the greatest number of eligible persons, which means offering vaccines at public places (e.g., markets, malls, churches), chiefdoms, workplaces, congregate settings, and others. 11. Using existing community health services for HIV as vaccination points. 12. Anticipating additional human resource needs and ensuring adequate financial resources to support them. **Demand generation:** 13. Ensuring adequate HCW training in HIV and other clinics to answer patients’ and eligible family members’ questions about COVID-19 vaccines. 14. Encouraging HCWs themselves to get vaccinated against COVID-19 by creating a safe space for unvaccinated HCWs to have their questions answered. 15. Engaging public and private media nationally to address myths and misconceptions about COVID-19 vaccines. 16. Developing promotional materials highlighting the importance of COVID-19 vaccination for people with HIV due to their higher risk of severe illness. 17. Engaging civil society leaders to champion COVID-19 vaccination and addressing their concerns through dialogue. 18. Using routine patient reminder call for upcoming visits to share information about vaccine availability in HIV clinics. **Monitoring and Evaluation (M&E)** 19. Harmonizing COVID-19 vaccine data collection in HIV and other clinics with the national COVID-19 vaccine M&E system. 20. Analyzing data frequently to inform site-level performance assessments and guiding targeted quality improvement. 21. Generating feedback loops, particularly for poorly performing districts. **Logistics:** 22. Pushing adequate vaccine supplies to each district based on their estimated target populations with the micro plan. 23. Taking inventory of health facility capacity to adequately store COVID-19 vaccines and using existing infrastructure where possible. 24. Ensuring that HIV clinic vaccine supply is incorporated into the wider health facility request **Safety:** 25. Providing Adverse Event Following Immunization (AEFI) training to HCWs. 26. Strengthening AEFI reporting system within HIV clinics.
Call-back strategy—an approach for scaling up COVID-19 vaccination among PLHIV in Geita region, Afya Jumuishi project	Magadula et al. [27]	2023	Tanzania	Not clear	44 facilities in Tanzania	PLHIV	Not reported	Call-back strategy	Oriented HCWs and Community Based Health Workers (CBHWs), invited PLHIV for facility-based vaccination, and conducted physical follow-ups.	**Increased number of PLHIV vaccinated:** a total of 22,686 PLHIV (83% from the shared list) were called and fully vaccinated. This contributed to 42% of total vaccinations of PLHIV.	Implementation innovations accelerate progress on program indicators, improve supply monitoring, and reduce resource wastage.

**Table 2:** Identified COVID-19 Vaccine Delivery Models.

Model	Details
**The Call-back Strategy**	In health service delivery, Call-back strategies refer to an act of calling patients to report back to a health facility for a specific intervention with the aim of improving patient outcomes, satisfaction, and operational efficiency [28]. In February 2022, Tanzania started a call-back strategy with health workers and community-based health workers to boost COVID-19 vaccination rates among PLHIV. The approach began by creating lists of 27,060 PLHIV on 3- and 6-month Multi-Month Dispensing (MMD) schedules. These lists were sent to 44 Tier 1 and Tier 2 health facilities. Facilities were responsible for retrieving patient records, inviting clients to come back for vaccination, and covering transportation costs for those who responded. For clients who could not be reached by the facility, community-based health workers, peer supporters, and counselors conducted follow-ups. By June 30th, 22,686 PLHIV, which is 83% of those listed, had been successfully contacted and fully vaccinated. This made up 42% of the total 53,286 PLHIV vaccinated as part of the overall campaign, achieving 91% of the target coverage. The strategy also allowed for the use of 8400 doses of the Moderna vaccine that were nearing expiration, helping to prevent wastage.
**The Diverse Partnership Model**	The diverse partnership model in health service delivery involves collaboration between multiple stakeholders, such as public and private sectors, and communities, to improve health outcomes, access, and efficiency [29]. Jalloh et al. [26] reported on a six-week COVID-19 vaccination effort aimed at people living with HIV and healthcare workers. This initiative took place in Tanzania from September 27 to November 7, 2021. It used a diverse partnership model that involved key stakeholders at both national and regional levels, including medical officers and commissioners. The model focused on the following key strategies: Training HCWs to increase the number of certified vaccinators. Setting up vaccination points in high-volume HIV clinics, prioritizing areas with strong political support and health system engagement. Engaging HCWs to build confidence and address concerns about the vaccine. Improving the skills of HCWs and community health workers to support vaccination in facilities and communities. Tracking vaccine uptake among PLHIV and HCWs, especially in high-volume facilities. As the initiative moved forward, core strategies were expanded in partnership with national immunization and HIV programs. This expansion was supported by joint supervision visits that provided real-time feedback and guidance to an extended network of facilities. The model also included a strategy to follow up PLHIV receiving multi-month dispensing (MMD) of Antiretroviral therapy (ART), encouraging them to return for vaccination. In certain facilities, transportation costs were covered to offset the expense of additional visits outside scheduled appointments. This approach played a key role in reducing vaccination disparities among PLHIV on MMD. COVID-19 educational materials were distributed in busy areas of facilities to raise vaccine awareness among patients and their families. Stakeholders reviewed the results and identified six areas for success and improvement: Collaboration and coordination, capacity building, data management, demand creation, supply chain and service delivery.
**Mixed Service Delivery Model**	A Mixed Service Delivery Model in health may refer to an approach that combines multiple methods, settings, or provider types to deliver healthcare services in a coordinated way to a population [30]. In Zambia, the authors described a mixed service delivery model that used strategies from existing childhood vaccination programs to improve COVID-19 vaccine uptake among people living with HIV. Key elements of this model included: Training HCWs to provide COVID-19 vaccination using existing human resources. Encouraging HCWs to get vaccinated first. This helped build trust and confidence in the vaccine. Creating targeted promotional materials specifically for PLHIV, who are at higher risk for severe COVID-19 illness. Quickly adapting and expanding these approaches nationwide. Building on the success in clinical settings, a community-based vaccination approach was added to existing static HIV community programs. Civil society leaders played a key role in supporting vaccination efforts, which were further intensified during a December campaign launched on World AIDS Day. This model effectively used HIV programs to aid COVID-19 vaccination efforts through six pillars: Planning and coordination, service delivery, demand creation, monitoring and evaluation, logistics and safety.

## Data Availability

The data used during the current study are available from the corresponding author on reasonable request. The data can be available in English for the readers and made available from the corresponding author on reasonable request.

## Appendix A. Search Strategy

**Table A1:** Detailed search strategy for Electronic Databases.

Database	Query Number	Query
	#4	#1 AND #2 AND #3
EMBASE Search Strategy	#3	(‘low-income countr*’ OR ‘middle-income countr*’ OR ‘developing countr*’ OR ‘low-resource countr*’ OR ‘resource-poor countr*’ OR ‘underdeveloped countr*’ OR ‘less developed countr*’ OR ‘global south’ OR ‘sub-saharan africa’/exp OR ‘sub-saharan africa’ OR ‘latin america’/exp OR ‘latin america’ OR ‘south asia’/exp OR ‘south asia’ OR ‘africa’/exp OR ‘africa’ OR ‘southeast asia’/exp OR ‘southeast asia’ OR ‘central america’/exp OR ‘central america’ OR ‘eastern europe’/exp OR ‘eastern europe’ OR ‘caribbean’/exp OR ‘caribbean’ OR ‘pacific islands’/exp OR ‘pacific islands’ OR ‘afghanistan’/exp OR afghanistan OR ‘angola’/exp OR angola OR ‘bangladesh’/exp OR bangladesh OR ‘benin’/exp OR benin OR ‘bhutan’/exp OR bhutan OR ‘bolivia’/exp OR bolivia OR ‘botswana’/exp OR botswana OR burkina) AND faso OR ‘burundi’/exp OR burundi OR…
	#2	‘HIV infections’/exp OR ‘HIV infections’ OR ‘HIV’/exp OR ‘HIV’ OR ‘HIV/AIDS’/exp OR ‘HIV/AIDS’ OR ‘people living with HIV’/exp OR ‘people living with HIV’ OR ‘PLHIV’ OR ‘HIV care’/exp OR ‘HIV care’ OR ‘HIV services’ OR ‘HIV treatment’:ti,ab
	#1	‘COVID-19 vaccines’/exp OR ‘COVID-19 vaccines’ OR ‘SARS-CoV-2 vaccine’/exp OR ‘SARS-CoV-2 vaccine’ OR ‘COVID-19 immunization’ OR ‘COVID-19 vaccination’:ti,ab
Cochrane Library Search Strategy	ID	Search Query
	#1	(“COVID-19 Vaccines” OR “SARS-CoV-2 vaccine” OR “COVID-19 immunization” OR “COVID-19 vaccination”):ti,ab,kw
	#2	(“HIV Infections” OR “HIV” OR “HIV/AIDS” OR “People Living with HIV” OR “PLHIV” OR “HIV Care” OR “HIV Services” OR “HIV Treatment”):ti,ab,kw
	#3	(((low-income NEXT countr*) OR (middle-income NEXT countr*) OR (developing NEXT countr*) OR (low-resource NEXT countr*) OR (resource-poor NEXT countr*) OR (underdeveloped NEXT countr*) OR (less NEXT developed NEXT countr*) OR (global NEXT south) OR (sub-Saharan NEXT Africa) OR (Latin NEXT America) OR (South NEXT Asia) OR (Africa) OR (Southeast NEXT Asia) OR (Central NEXT America) OR (Eastern NEXT Europe) OR (Caribbean) OR (Pacific NEXT Islands)) OR (Afghanistan OR Angola OR Bangladesh OR Benin OR Bhutan OR Bolivia OR Botswana OR Burkina NEXT Faso OR Burundi OR Cambodia OR Cameroon OR Central NEXT African NEXT Republic OR Chad OR Comoros OR Congo OR Democratic NEXT Republic NEXT of NEXT the NEXT Congo OR Djibouti OR Egypt OR El NEXT Salvador OR Eritrea OR Eswatini OR Ethiopia OR Fiji OR Gambia OR Ghana OR Guatemala OR Guinea OR Guinea-Bissau OR Haiti OR Honduras OR India OR Indonesia OR Kenya OR Kiribati OR Korea, NEXT North OR Kyrgyzstan OR Laos OR Lesotho OR Liberia OR Madagascar OR Malawi OR Mali OR Mauritania OR Micronesia OR Mongolia OR Mozambique OR Myanmar OR Nepal OR Nicaragua OR Niger OR Nigeria OR Pakistan OR Papua NEXT New NEXT Guinea OR Philippines OR Rwanda OR Samoa OR Senegal OR Sierra NEXT Leone OR Solomon NEXT Islands OR Somalia OR South NEXT Sudan OR Sudan OR Syria OR Tajikistan OR Tanzania OR Timor-Leste OR Togo OR Tonga OR Turkmenistan OR Uganda OR Uzbekistan OR Vanuatu OR Venezuela OR Vietnam OR Yemen OR Zambia OR Zimbabwe)):ti,ab,kw
	#4	#1 AND #2 AND #3
Web of Science Search Strategy	ID	Search Query
	1	TS = (“COVID-19 Vaccines” OR “SARS-CoV-2 vaccine” OR “COVID-19 immunization” OR “COVID-19 vaccination”)
	2	TS = (“HIV Infections” OR “HIV” OR “HIV/AIDS” OR “People Living with HIV” OR “PLHIV” OR “HIV Care” OR “HIV Services” OR “HIV Treatment”)
	3	TS = (“Health Services Integration” OR “Integrated Care” OR “Service Delivery Models” OR “Integration Models” OR “Health System Integration” OR “Health Services Delivery”)
	4	TS = (“low-income countr*” OR “middle-income countr*” OR “developing countr*” OR “low-resource countr*” OR “resource-poor countr*” OR “underdeveloped countr*” OR “less developed countr*” OR “global south” OR “sub-Saharan Africa” OR “Latin America” OR “South Asia” OR “Africa” OR “Southeast Asia” OR “Central America” OR “Eastern Europe” OR “Caribbean” OR “Pacific Islands” OR Afghanistan OR Angola OR Bangladesh OR Benin OR Bhutan OR Bolivia OR Botswana OR Burkina Faso OR Burundi OR Cambodia OR Cameroon OR Central African Republic OR Chad OR Comoros OR Congo OR “Democratic Republic of the Congo” OR Djibouti OR Egypt OR El Salvador OR Eritrea OR Eswatini OR Ethiopia OR Fiji OR Gambia OR Ghana OR Guatemala OR Guinea OR Guinea-Bissau OR Haiti OR Honduras OR India OR Indonesia OR Kenya OR Kiribati OR “Korea, North” OR Kyrgyzstan OR Laos OR Lesotho OR Liberia OR Madagascar OR Malawi OR Mali OR Mauritania OR Micronesia OR Mongolia OR Mozambique OR Myanmar OR Nepal OR Nicaragua OR Niger OR Nigeria OR Pakistan OR “Papua New Guinea” OR Philippines OR Rwanda OR Samoa OR Senegal OR “Sierra Leone” OR “Solomon Islands” OR Somalia OR “South Sudan” OR Sudan OR Syria OR Tajikistan OR Tanzania OR Timor-Leste OR Togo OR Tonga OR Turkmenistan OR Uganda OR Uzbekistan OR Vanuatu OR Venezuela OR Vietnam OR Yemen OR Zambia OR Zimbabwe)
	5	#1 AND #2 AND #4
PubMed Search Strategy	Search number	Query
	6	((“COVID-19 Vaccines” OR “SARS-CoV-2 vaccine” OR “COVID-19 immunization” OR “COVID-19 vaccination”) AND (“HIV Infections” OR “HIV” OR “HIV/AIDS” OR “People Living with HIV” OR “PLHIV” OR “HIV Care” OR “HIV Services” OR “HIV Treatment”)) AND (“low-income countr*” OR “middle-income countr*” OR “developing countr*” OR “low-resource countr*” OR “resource-poor countr*” OR “underdeveloped countr*” OR “less developed countr*” OR “global south” OR “sub-Saharan Africa” OR “Latin America” OR “South Asia” OR “Africa” OR “Southeast Asia” OR “Central America” OR “Eastern Europe” OR “Caribbean” OR “Pacific Islands” OR Afghanistan OR Angola OR Bangladesh OR Benin OR Bhutan OR Bolivia OR Botswana OR Burkina Faso OR Burundi OR Cambodia OR Cameroon OR Central African Republic OR Chad OR Comoros OR Congo OR “Democratic Republic of the Congo” OR Djibouti OR Egypt OR El Salvador OR Eritrea OR Eswatini OR Ethiopia OR Fiji OR Gambia OR Ghana OR Guatemala OR Guinea OR Guinea-Bissau OR Haiti OR Honduras OR India OR Indonesia OR Kenya OR Kiribati OR Korea, North OR Kyrgyzstan OR Laos OR Lesotho OR Liberia OR Madagascar OR Malawi OR Mali OR Mauritania OR Micronesia OR Mongolia OR Mozambique OR Myanmar OR Nepal OR Nicaragua OR Niger OR Nigeria OR Pakistan OR Papua New Guinea OR Philippines OR Rwanda OR Samoa OR Senegal OR Sierra Leone OR Solomon Islands OR Somalia OR South Sudan OR Sudan OR Syria OR Tajikistan OR Tanzania OR Timor-Leste OR Togo OR Tonga OR Turkmenistan OR Uganda OR Uzbekistan OR Vanuatu OR Venezuela OR Vietnam OR Yemen OR Zambia OR Zimbabwe)
	5	“low-income countr*” OR “middle-income countr*” OR “developing countr*” OR “low-resource countr*” OR “resource-poor countr*” OR “underdeveloped countr*” OR “less developed countr*” OR “global south” OR “sub-Saharan Africa” OR “Latin America” OR “South Asia” OR “Africa” OR “Southeast Asia” OR “Central America” OR “Eastern Europe” OR “Caribbean” OR “Pacific Islands” OR Afghanistan OR Angola OR Bangladesh OR Benin OR Bhutan OR Bolivia OR Botswana OR Burkina Faso OR Burundi OR Cambodia OR Cameroon OR Central African Republic OR Chad OR Comoros OR Congo OR “Democratic Republic of the Congo” OR Djibouti OR Egypt OR El Salvador OR Eritrea OR Eswatini OR Ethiopia OR Fiji OR Gambia OR Ghana OR Guatemala OR Guinea OR Guinea-Bissau OR Haiti OR Honduras OR India OR Indonesia OR Kenya OR Kiribati OR Korea, North OR Kyrgyzstan OR Laos OR Lesotho OR Liberia OR Madagascar OR Malawi OR Mali OR Mauritania OR Micronesia OR Mongolia OR Mozambique OR Myanmar OR Nepal OR Nicaragua OR Niger OR Nigeria OR Pakistan OR Papua New Guinea OR Philippines OR Rwanda OR Samoa OR Senegal OR Sierra Leone OR Solomon Islands OR Somalia OR South Sudan OR Sudan OR Syria OR Tajikistan OR Tanzania OR Timor-Leste OR Togo OR Tonga OR Turkmenistan OR Uganda OR Uzbekistan OR Vanuatu OR Venezuela OR Vietnam OR Yemen OR Zambia OR Zimbabwe
	3	(“COVID-19 Vaccines” OR “SARS-CoV-2 vaccine” OR “COVID-19 immunization” OR “COVID-19 vaccination”) AND (“HIV Infections” OR “HIV” OR “HIV/AIDS” OR “People Living with HIV” OR “PLHIV” OR “HIV Care” OR “HIV Services” OR “HIV Treatment”)
	4	“Health Services Integration” OR “Integrated Care” OR “Service Delivery Models” OR “Integration Models” OR “Health System Integration” OR “Health Services Delivery”
	2	“HIV Infections” OR “HIV” OR “HIV/AIDS” OR “People Living with HIV” OR “PLHIV” OR “HIV Care” OR “HIV Services” OR “HIV Treatment”
	1	“COVID-19 Vaccines” OR “SARS-CoV-2 vaccine” OR “COVID-19 immunization” OR “COVID-19 vaccination”
